# Use of Continuous Glucose Monitoring in Older Adults: A Review of Benefits, Challenges and Future Directions

**DOI:** 10.17925/EE.2022.18.2.116

**Published:** 2022-11-30

**Authors:** Lalita Prasad-Reddy, Alvin Godina, Ashwin Chetty, Diana Isaacs

**Affiliations:** 1. Chicago Medical School, Rosalind Franklin University of Medicine and Science, Chicago, IL, USA; 2. Adult Internal Medicine, Rush University Internists, Chicago, IL, USA; 3. Ambulatory Care, Rush University Medical Center, Chicago, IL, USA; 4. Close Concerns, San Francisco, CA, USA; 5. Cleveland Clinic Endocrinology & Metabolism Institute, Cleveland, OH, USA

**Keywords:** Diabetes, continuous glucose monitoring, diabetes technology, older adults, blood glucose monitoring, diabetes management

## Abstract

Many new technologies have been developed over the past decade, and these have substantially changed the way diabetes is managed. Continuous glucose monitoring is now the standard of care for many people living with diabetes, and among its numerous benefits, it has been shown to improve glycaemic outcomes and enhance quality of life. Older adults carry a high burden of diabetes and have a high risk of hypo-glycaemia and hypo-glycaemic unawareness, and continuous glucose monitoring can help to improve glycaemic management in this vulnerable population. Unfortunately, only a few trials have evaluated the effectiveness of continuous glucose monitoring in older adults. Certainly, the implementation of continuous glucose monitoring in older adults can come with many challenges, including logistical, educational and reimbursement barriers. This article will discuss the benefits of continuous glucose monitoring in older adults with diabetes, the clinical studies that support its use and the barriers to its optimal implementation in this population.

Recent advances in technology have changed the landscape for managing diabetes treatment. Thanks to innovations such as connected insulin pens, sensor-augmented pump systems, automated insulin delivery, integrated mobile applications and continuous glucose monitoring (CGM) systems, people with diabetes now have access to devices that are easier to use and far less invasive than those available previously. Although CGM is now a standard of care for people living with diabetes, its use – especially in older populations – is under-appreciated. The benefit of CGM in adults with diabetes has been repeatedly shown to improve glycaemic outcomes and quality of life.^1–6^ Unfortunately, many clinical trials included either small proportions of older adults or none at all. Thus, their results are difficult to extrapolate to this population subset. More recently, new data and guidelines have been published that specifically address the use of diabetes technology in older adults.

CGM systems measure glucose in the interstitial fluid every 1–5 minutes and record this information every 5–15 minutes, depending on the device. Many CGM devices offer alerts for customizable high or low glucose thresholds. Furthermore, many devices can predict when glucose will reach such a threshold and provide alerts, which can be beneficial in older adults.^7^ For some devices, the transmitter and sensor are connected (e.g. FreeStyle Libre [Abbott Laboratories, Chicago, IL, USA]),^8,9^ whereas others require an additional step to attach the transmitter (Dexcom G6 [Dexcom, San Diego, CA, USA], Eversense® [Ascensia, Basel, Switzerland], Guardian™ [Medtronic, Dublin, Ireland]).^10–13^ A reader, receiver or mobile application displays the glucose readings and trend arrows and indicates whether glucose is rising or falling. Many devices are approved by the US Food and Drug Administration (FDA) to replace blood glucose monitoring, also called a non-adjunctive indication.^14^ A key difference between systems is their ability to integrate with connected insulin pens and insulin pumps. *Table 1* compares the CGM systems available in the USA.

## Guideline overview for older adults

The 2021 American Association of Clinical Endocrinology (AACE) Clinical Practice Guideline for the use of technology in diabetes suggests that CGM in older adults reduces haemoglobin A1c (HbA1c), improves quality of life, and improves the detection – and reduces the incidence – of hypo-glycemia.^15–19^ Further, the 2022 American Diabetes Association (ADA) Standards of Medical Care in Diabetes recommends that CGM be considered to reduce hypo-glycaemia in older adults with type 1 diabetes and that it may have a significant role for older adults with cognitive or physical impairments or in those whose glucose monitoring is carried out by a caregiver.^20^

**Table 1:** Available continuous glucose monitoring systems^8–14^

	Dexon G6	Libre 2	Libre 3	Guardian™ Connect or Guardian™ 3	Eversense®
Integration	T:Slim X2 (Tandem Diabetes Care, San Francisco, CA, USA) Omnipod 5 (Insulet, Acton, MA, USA) InPen (Medtronic Diabetes, Northridge, CA, USA)	Bigfoot Unity (Bigfoot, Milpitas, CA, USA)	None	Medtronic 770G (Medtronic Diabetes, Northridge, CA, USA) InPen (Medtronic Diabetes, Northridge, CA, USA)	None
Display device	Smartphone or receiver or insulin pump	Smart phone or reader	Smartphone	Smartphone or insulin pump	Smartphone
Maximum wear time	10 days	14 days	14 days	7 days	180 days
Warm-up time	2 hours	1 hour	1 hour	Up to 2 hours	24 hours
Calibrations required	0	0	0	At least two per day	Two per day for 21 days, then once per day
FDA-approved sites	Abdomen Upper buttocks	Upper arm	Upper arm	Upper arm, abdomen Upper buttocks	Upper arm
FDA approved for dosing without confirmatory fingersticks	Yes	Yes	Yes	No	Yes
FDA-approved ages (years)	≥2	≥4	≥4	≥2 Guardian 3 ≥14 Guardian Connect	≥18
Drug interactions	Hydroxyurea	Vitamin C	Vitamin C	Acetaminophen Hydroxyurea	Tetracycline antibiotics Mannitol
MARD	9%	9.2%	7.9%	9.64%	8.5%
Alarms	High Low Predictive	High Low	High Low	High Low Predictive	High Low Predictive

*FDA = US Food and Drug Administration; MARD = Mean absolute relative difference between sensor glucose and venous YSI measurements*

**Table 2:** American Diabetes Association glucose targets^22^

Health status	HbA1c goal, %	Fasting glucose, mg/dL	Bedtime glucose, mg/dL
Healthy: Few co-existing chronic illnesses, intact cognitive and functional status	<7.0–7.5	80–130	80–180
Complex/intermediate: Multiple co-existing chronic illnesses, two or more instrumental daily living impairments, or mild-to-moderate cognitive impairment	<8.0	90–150	100–180
Very complex/poor: Long-term care, end-stage chronic illness, moderate-to-severe cognitive impairment, or two or more daily living impairments	Avoid hypo-glycaemia and symptomatic hyper-glycaemia; do not rely on HbA1c	100–180	110–200

*HbA1c = haemoglobin A1c*

Despite these mentions in recent guidelines, the appropriate usage of CGM in older adults remains largely undefined. While the ADA recommends the use of CGM in adults taking multiple daily injections or continuous insulin infusion, and states that CGM can be used in adults on basal insulin,^21^ CGM is not specifically recommended for use in older adults with type 2 diabetes taking insulin.^20^

The ADA suggests that, in the future, CGM should play a role in the treatment of older adults who have physical or cognitive limitations that hinder their ability to self-monitor blood glucose. Further, the ADA recommends modified glycaemic targets for older adults depending on an individual’s health status, which is defined as ranging from ‘healthy’, to ‘complex’ to ‘very complex/poor’ (*Table 2*).^22^

In contrast to the ADA, the AACE recommends real-time CGM for people aged 65 years and over with insulin-dependent diabetes, with an ultimate goal of improving glycaemic control, reducing severe hypoglycaemia and improving quality of life.^15^ Due to the increased risk of severe hypo-glycaemia and reduced capacity for detection in older adults, the AACE also recommends individualized glycemic targets for older adults, and further suggests less stringent thresholds specific for CGM.^15^ Similarly, the international consensus on time in range defines less stringent targets for older adults, and these have been adopted by the ADA. (*Table 3*).^23^

**Table 3:** International consensus on time in range^23^

	% time in range, 70–180 mg/dL	% time below range, <70 mg/dL	% time below range, <54 mg/dL	% time above range, >180 mg/dL	% time above range, >250 mg/dL
Older/high risk T1D/T2D	>50	<1	∼0	<50	<10
General T1D/T2D	>70	<4	<1	<25	<5

*T1D = type 1 diabetes; T2D = type 1/2 diabetes.*

## Benefits of continuous glucose monitoring in older adults

The potential benefits of CGM in older adults are widespread and include reduction in hyper-glycaemia and hypo-glycaemia, improvement in quality of life and prevention of diabetes-related complications.^15^ However, the evidence base is small because only a few studies have examined the use of CGM in older adults, and its application is likely to be under-appreciated. In one analysis, less than 40% of people with diabetes were using diabetes technology and even fewer of patients aged over 50 years,^24^ a population that can uniquely benefit from the use of diabetes technology.

## Hypo-glycaemia, older adults and continuous glucose monitoring

The frequency of hypo-glycaemia reported in the literature varies, often due to the criteria used to define a hypo-glycaemic episode. Yet, in general, hypo-glycaemic events tend to occur more frequently among older populations compared with younger ones. In one study, older people (≥70 years) reported more episodes than younger people (<60 years) (12.8% versus 9.0%; p<0.01). Significant differences were also seen for symptomatic episodes without a need for help (9.2% versus 5.6%).^25^ Additionally, retrospective analysis of annual hospitalizations due to hypo-glycaemic events also follows a similar trend: the rate among older individuals is double that of younger patients.^26^

While it is well documented that older adults are more prone to hypoglycaemia and hypo-glycaemic unawareness, the severe consequences of hypo-glycaemia in older adults is under-appreciated.^25^ Severe hypoglycaemia has serious consequences and can result in substantial morbidity, including cardiac abnormalities, seizures or even death. Even more troubling is that older adults with long-standing disease – such as those living with type 1 diabetes – are at a higher risk of death when hypo-glycaemia does occur.^27,28^ While mild or moderate hypo-glycaemia is more likely to be under-reported than more severe episodes, frequent and progressive events that occur due to hypoglycaemia unawareness has been associated with physical and mental disability.^25^ The cyclical nature of hypo-glycaemic unawareness poses a significant risk to the older population because of its associated negative outcomes: these include falls, incontinence, frailty, cognitive impairment or depressive symptoms.^29^

Hypo-glycaemia is associated with broad-ranging and numerous consequences, and these can impact quality of life. For example, the risk of falls among older patients who experience hypo-glycaemia is twice that of younger patients experiencing hypo-glycemia.^25^ Further, hypoglycemia can lead to patients avoiding certain activities due to a decline in ability, real or perceived, to perform physical tasks. This decline in physical function increases anxiety and social isolation. Hypo-glycaemic events also negatively impact cognition, with three or more events increasing the likelihood of a subsequent diagnosis of dementia.^30^

Minimizing hypo-glycaemia is a treatment goal for older adults living with diabetes. As mentioned earlier, the ADA recommends relaxed HbA1c treatment goals for older patients or for those with a history of frequent hypo-glycaemic episodes.^22^ While this measure aims to prevent hypoglycaemia, some studies suggest that older adults with HbA1c glucose targets lower than 8% exhibit the same degree of hypo-glycaemia as those with even lower HbA1c targets.^31^ It has also been postulated that HbA1c is not a strong predictor of hypo-glycaemia, and can reflect a wide range of glucose concentrations.^22^

While CGM has been shown to reduce the risk of hypo-glycaemia in people with diabetes in several trials, its use in the older population is largely based upon anecdotal and extrapolated data. A few well-conducted studies have outlined the potential benefits of CGM in older patients and should not be overlooked.

## Clinical studies of continuous glucose monitoring in older adults

The DIAMOND study was a large, randomized controlled trial that compared the use of CGM with self-monitoring of blood glucose in people living with diabetes and taking multiple daily insulin injections.^1^ The original trial included a large age span (26–79 years) and showed that the use of CGM was associated with a significantly greater decrease in HbA1c when compared with self-monitoring of blood glucose. A sub-analysis of the trial evaluated the potential benefit of CGM in older adults (those aged >60 years) living with either type 1 or type 2 diabetes, with an HbA1C of 7.5–10% and on multiple daily doses of insulin. Within the original trial, 116 people met the criteria for the sub-analysis. At the end of the 24-week treatment period, patients in the CGM group experienced significantly greater reductions in HbA1c compared with the controls (-0.9 versus -0.5, respectively [adjusted difference in mean change was -0.4, p<0.001]). Additionally, use of CGM was associated with significantly less time, with blood glucose of >250 mg/dL and lower glycaemic variability. The trial originally sought to compare rates of hypo-glycaemia between treatment arms, but rates were too low in both groups to detect any meaningful difference. In addition to the clinical outcomes, the participants were also asked to complete the CGM satisfaction survey, and the results were positive overall: individuals who used CGM felt it to be beneficial and causing little hassle.^18^

Pratley et al. conducted another ground-breaking study of CGM in older adults.^19^ The WISDM study evaluated the potential benefits of CGM in older adults with type 1 diabetes. Those included were at least 60 years of age, with HbA1c levels <10% and using either a subcutaneous insulin pump or taking multiple daily insulin injections. Individuals were randomized to use either CGM or standard blood glucose monitoring. The primary outcome was the time spent in hypo-glycaemia, defined as blood glucose <70 mg/ dL. At the end of the 6-month treatment period, individuals randomized to the CGM group spent slightly but significantly less time in hypo-glycaemia than the standard group (2.7% [39 min/day] versus 4.9% [70 min/day]; adjusted treatment difference, -1.9% (-27 min/day); 95% confidence interval [CI] -2.8% to -1.1% [-40 to -16 min/day]; p<0.001). Overall, this translated to a reduction in overall hypo-glycaemia by approximately 27 minutes per day. Subset analyses showed that individuals who had higher glycaemic variability and baseline risk of hypo-glycaemia had a more profound treatment effect. It should be noted that while CGM usage in this trial associated with a small decrease in hypo-glycaemia, most people were still unable to achieve the treatment goal for older adults, which is less than 1% of time spent with blood glucose <70 mg/dL.^19^

Researchers of the WISDM trial extended the study to an observational extension phase for an additional 6 months. The objective of the extension was to determine whether the glycaemic improvements seen in people who used CGM during the randomized part of the trial were sustained over time. The extension trial followed 100 participants originally randomized to the CGM group, and 94 participants originally randomized to conventional blood glucose monitoring, and implemented CGM in both cohorts. At the end of the 6-month treatment period, participants who remained on CGM for both the original and extension phases of the study had sustained reductions in time spent in hypo-glycaemia (p<0.001 baseline to 52 weeks), continued to experience benefits in time in range (mean 56% at baseline to 64% at 52 weeks; p<0.001), and had significantly reduced HbA1c levels at 52 weeks compared with baseline (mean 7.6% [59 mmol/mol] versus 7.4% [57 mmol/mol]; p=0.01). In the group that had transitioned from blood glucose monitoring to CGM, the time spent in hypo-glycaemia was significantly reduced over the course of the extension treatment period (3.9% to 1.9%, [p<0.001]). Similarly, time in range increased significantly from 56% to 60% (p=0.006) and HbA1c decreased significantly from 7.5% (58 mmol/mol) to 7.3% (57 mmol/mol; p=0.025). The authors concluded that CGM should be considered as a standard of care for older adults living with type 1 diabetes, given their hypo-glycaemic vulnerability.^32^

MOBILE was an 8-month randomized trial that compared the use of CGM with conventional blood glucose monitoring in people living with type 2 diabetes on basal insulin.^33^ A subgroup analysis compared the treatment effect in older adults (65–79 years) with those in younger adults (<65 years). For participants aged 65 years or older, the change from baseline in HbA1c was -1.08% in the CGM group and -0.38% in the conventional blood glucose monitoring group (adjusted mean difference: -0.65% [95% CI -1.49 to 0.19]). In contrast, the change in HbA1c between treatment groups was -0.35% [95% CI -0.77 to 0.07] in the <65 years age group. For time spent in hyper-glycaemia, treatment differences favoured the CGM group in both age groups. For time in range 70–180 mg/dL, the mean adjusted treatment group difference was 19% (95% CI 4–35; p=0.01) in the older age group and 12% (95% CI 4–19, p=0.003) in the younger age group.^33^ Overall, this study supports the advantage of CGM compared with conventional blood glucose monitoring in older adults on basal insulin and showed various other favourable outcomes including improved time in range and reduction in hyper-glycaemia.

## Patient satisfaction and quality of life measures

The feasibility of CGM is a concern in older adults. Challenges with technology, calibration and technical aspects can limit a peron’s confidence in their ability to successfully manage CGM. Nonetheless, several studies have shown that CGM can deliver benefits in older adults with diabetes, in the form of enhanced empowerment and patient satisfaction, while allaying fears of hypo-glycaemia without causing significant barriers or annoyances.

In a small feasibility study, six individuals with diabetes who were aged 75 years or over, on insulin therapy and managing their disease at home, were asked to wear a CGM device at their home for 5 days, and answer standardized questionnaires about their overall satisfaction, ease of use, and device acceptability. Older adults with severe cognitive challenges, such as severe dementia, were excluded. All six participants completed the study. Overall, CGM was found to be feasible and accurately predicted concerns over hypo-glycaemia.^34^

Another study evaluated patient-reported outcomes relating to CGM in older individuals on insulin therapy with well-managed diabetes. Twenty-five adults aged over 65 years were instructed to wear a CGM device and answer questionnaires on treatment satisfaction. Overall, CGM was associated with high treatment satisfaction. Participants perceived the advantages of CGM to be very high and the annoyances to be modest. The vast majority reported an improved sense of security while wearing the CGM and did not report the device to impose additional diabetes-related distress. CGM metrics also had substantial improvements including improving time in range (p<0.001) and reducing the time spent in hypo-glycaemia (p=0.041).^35^ This study demonstrated that wearing a CGM device can enhance the treatment satisfaction of older adults living with diabetes, without imposing significant stress or distress. Of note, people in this study had generally well-controlled diabetes (with HbA1c around 7.2%), so it is challenging to extrapolate these study results to older adults, who present with large glycaemic variabilities or significantly elevated HbA1c levels.^35^

Many individuals may be able to experience the benefits of CGM before they reach the age of 65 years. Upon retirement in the USA, people with diabetes are at risk of losing access to the technology. Because of this risk, the ADA warns that individuals who have previously had access to a real-time CGM device must have continued access even after they reach 65 years of age.^21^ A study surveyed adults over 65 years who were seeking use of a CGM and analysed the results from two cohorts: those who were successful in obtaining a device and currently using one and those who were unsuccessful in obtaining a device (known in the study as CGM-hopefuls). Compared with those who had secured and were using a CGM device, the hopeful cohort reported having a significantly lower income and significantly lesser education. Those were the underlying root-causes of the disparity, with evident differences in access to diabetes technology according to socioeconomic status. Compared with those using CGM devices, people in the hopeful cohort also reported more hypo-glycaemic episodes and significantly more interventions requiring emergency room visits or paramedic home visits. Furthermore, overall wellbeing was reduced in the hopeful group, evidenced by increased diabetes-related distress, fear of hypo-glycaemia and feelings of powerlessness. This study demonstrated that, as well as improving glycaemic metrics, the use of CGM technology could also greatly improve quality of life.^16^

Many older adults have caregivers and family members as integral members of their diabetes care team. Newer smartphone apps can alert external parties to CGM values and data. Allen et al. evaluated a sharing data intervention between older adults living with type 1 diabetes and their caregivers. The sharing data intervention was associated with high satisfaction among patients and caregivers, as well as with improved quality of life. Most individuals perceived a benefit from the support they received arising from data sharing, although a minority of participants (15%), felt that care caregivers over-reacted to available data. Still, this study showed the benefit that CGM can provide not only to patients but also to caregivers to improve support and enhance problem solving.^36^

## Barriers to successful continuous glucose monitoring implementation

CGM systems rely on devices with multiple components, which are worn for variable lengths of time. Although exceptions within specific insurance plans exist, in today’s practice in the USA coverage of a CGM system is often dictated by requirements set by the Centers for Medicare and Medicaid Services. These requirements have included frequent adjustments of insulin regimen, the need for frequent blood glucose testing (≥4 tests/day), and use of an insulin pump or three or more insulin injections per day.^37^ Over time, the requirements have been relaxed, and most recently the need for ≥4 glucose tests per day was eliminated. During the COVID-19 public health emergency, the specific indications for the use and coverage of CGM systems were relaxed among certain payers. Given the benefits associated with use of CGM devices, calls for improved coverage have led to a change in the legislation.^37^ One such example is a law signed in Illinois, USA, which requires insurance plans to provide coverage of CGM devices by 2024.^38^

While it is clear that minimizing hypo-glycaemia is a key treatment goal for older adults, it is unclear how best to use CGM for older adults with very complex, late-stage disease. Unfortunately, self-management of hypoglycaemia is challenging for older adults, as evidence suggests that they have more challenges with problem-solving when hypo-glycaemia does occur.^39^ People who are frail or have dementia have further limitations that may make optimal CGM implementation even more challenging.^39^ Although data are scarce, a feasibility study has been done that looked at implementation of CGM in the community setting in older adults with memory problems or dementia. Although some participants did report on-demand scanning to be challenging with intermittently scanned CGM, realtime devices with automated transfer were highly acceptable, improving usability and metrics over traditional blood glucose monitoring.^39^

CGM devices generate a large amount of data, and this can be a barrier to use. As we have seen, problem solving to treat hyper- and hypoglycaemia can be a challenging for older adults, as can the constant burden of interpreting and addressing high or low readings from their CGM device along with trend arrows indicating the direction and speed glucose is changing. Furthermore, the complexity of the monitoring systems and sensor codes can be a barrier when setting up mobile applications and changing the transmitter. Dexterity challenges, or visual or hearing impairments, are also common in older adults, so manually using the device and hearing its alarms may also present limitations.^40^ Therefore, extra attention must be paid to ensure that these barriers – specific to older people – have been addressed to maximize the potential benefits that CGM devices can deliver to older adults.

While extra attention must be paid to reducing these barriers for older adults, this is not always easy in practice. The need for increased training can pose a significant challenge to clinicians, who do not have the time or resources to train older adults, their caregivers, family members and others who play a role in the person’s care.^41^ Nevertheless, once trained, older adults demonstrate high rates of CGM wear and use, as evidenced by the WISDM trial.^19,32^ Still, providers should individualize the implementation of CGM in older adults with diabetes, and should assess individuals’ barriers, abilities and preferences when deciding when and how to incorporate CGM into their daily management.

## Role of the healthcare professional and future directions

Successful implementation of CGM devices requires understanding the technology, which in turn allows for the best use of the various products available in clinical practice. Various considerations exist for choosing the best product for a patient. These include their ability or willingness to calibrate the device, the need for a device with alarms or integration with an insulin pump.^42^ As the advances in technology are continually evolving, yet often subtle, healthcare professional must stay up to date and serve as a reference for patients and providers. The identify, configure, collaborate (ICC) framework can help overcome barriers to starting CGM by identifying the right technology, for the right person, at the right time.^43^ Subsequently, healthcare professionals can assist with device configuration based on user preferences, including setting optimal alarms and reminders, and then collaborate with the person to review the data and modify their treatment plan as needed through shared decision-making.^43^

Education for older adults using CGM should include discussion on many topics, including the reliability of the data, interpretation of results, drug interactions and the CGM devices themselves. People benefit from a thorough introduction on how to use the device safely, understand its alarms, how to interpret its values, and on its safe disposal. For individuals with caregivers, they too should be educated appropriately. As we have seen, studies have demonstrated high patient and caregiver satisfaction when a data-sharing intervention is employed.^36^

As mentioned earlier, the global implementation of CGM is often limited by cost. Certainly in the USA, where payers dictate product coverage, insurance is a consideration when selecting the optimal CGM device for a person to use. Ensuring people with diabetes are able to afford the product selected and its ongoing usage costs is crucial to continued use, and can be a challenge to older adults with fixed incomes. Although insurance coverage of CGM largely follows the coverage criteria set out by the Centers for Medicare and Medicaid Services, there are exceptions. Knowing these exceptions may help healthcare providers to broaden the pool of patients who may benefit from use of these devices. Unfortunately, there continues to be a lack of cost-effectiveness data on CGM use in older adults. Having these data could improve the prescription and uptake of CGM in this vulnerable population.

## Conclusion

CGM offers users a more holistic view of glucose management, incorporating time in range and glucose variability. It also has benefits regarding ease of use by reducing fingersticks. Caregivers and family members can remotely view the data with many devices. Benefits include improved time in range, reduced time in hyper- and hypo-glycaemia, and improved patient satisfaction. Unfortunately, the optimal implementation of CGM in older adults continues to be largely undefined, and various barriers limit their widespread use in this population: these include insurance coverage for devices (in the USA), requirements for education and logistical challenges for implementation. Healthcare professionals should take an individualized approach to identify older adults who may benefit from CGM technology and work on processes to streamline education, training and follow-up.
